# *Mycobacterium tuberculosis* Catalase Inhibits the Formation of Mast Cell Extracellular Traps

**DOI:** 10.3389/fimmu.2018.01161

**Published:** 2018-05-28

**Authors:** Marcia Campillo-Navarro, Kahiry Leyva-Paredes, Luis Donis-Maturano, Gloria M. Rodríguez-López, Rodolfo Soria-Castro, Blanca Estela García-Pérez, Nahum Puebla-Osorio, Stephen E. Ullrich, Julieta Luna-Herrera, Leopoldo Flores-Romo, Héctor Sumano-López, Sonia M. Pérez-Tapia, Sergio Estrada-Parra, Iris Estrada-García, Rommel Chacón-Salinas

**Affiliations:** ^1^Departamento de Inmunología, Escuela Nacional de Ciencias Biológicas, Instituto Politécnico Nacional, ENCB-IPN, México City, Mexico; ^2^Departamento de Fisiología y Farmacología, Facultad de Medicina Veterinaria y Zootecnia, Universidad Nacional Autónoma de México, UNAM, México City, Mexico; ^3^Department of Cell Biology, Cinvestav, Instituto Politécnico Nacional, México City, Mexico; ^4^Department of Lymphoma and Myeloma, The University of Texas MD Anderson Cancer Center, Houston, TX, United States; ^5^Department of Immunology, The Center for Cancer Immunology Research, The University of Texas MD Anderson Cancer Center, Houston, TX, United States; ^6^The University of Texas Graduate School of Biological Sciences at Houston, Houston, TX, United States; ^7^Unidad de Desarrollo e Investigación en Bioprocesos (UDIBI), Escuela Nacional de Ciencias Biológicas, Instituto Politécnico Nacional, ENCB-IPN, México City, Mexico

**Keywords:** tuberculosis, *Mycobacterium tuberculosis*, mast cell, mast cell extracellular trap, catalase

## Abstract

Tuberculosis is one of the leading causes of human morbidity and mortality. *Mycobacterium tuberculosis* (Mtb) employs different strategies to evade and counterattack immune responses persisting for years. Mast cells are crucial during innate immune responses and help clear infections *via* inflammation or by direct antibacterial activity through extracellular traps (MCETs). Whether Mtb induce MCETs production is unknown. In this study, we report that viable Mtb did not induce DNA release by mast cells, but heat-killed Mtb (HK-Mtb) did. DNA released by mast cells after stimulation with HK-Mtb was complexed with histone and tryptase. MCETs induced with PMA and HK-Mtb were unable to kill live Mtb bacilli. Mast cells stimulated with HK-Mtb induced hydrogen peroxide production, whereas cells stimulated with viable Mtb did not. Moreover, MCETs induction by HK-Mtb was dependent of NADPH oxidase activity, because its blockade resulted in a diminished DNA release by mast cells. Interestingly, catalase-deficient Mtb induced a significant production of hydrogen peroxide and DNA release by mast cells, indicating that catalase produced by Mtb prevents MCETs release by degrading hydrogen peroxide. Our findings show a new strategy employed by Mtb to overcome the immune response through inhibiting MCETs formation, which could be relevant during early stages of infection.

## Introduction

*Mycobacterium tuberculosis* (Mtb) is one of the most important pathogens affecting human health worldwide. The World Health Organization estimates that one-third of the human population is infected with this bacterium and approximately 5–10% of infected persons will develop a clinical manifestation of the infection (1).

*Mycobacterium tuberculosis* is an intracellular bacillus that has acquired different mechanisms to evade the immune response to survive and persist in the host. Mtb gains access to the host through the airways and reaches lung alveoli, where it interacts with different cells of the innate immune response (2). These cells recognize Mtb through different pattern-recognition receptors leading to the activation of different antimicrobial mechanisms (3). Phagocytosis is traditionally considered as one of the first mechanisms used by the host immune response. Macrophages, neutrophils, and dendritic cells have been identified as cells that phagocytose Mtb bacilli; however, elimination of the infection is usually not achieved (4). To this end, Mtb deploy different mechanisms to evade its killing in phagocytic cells, such as inhibiting phagosome maturation (5), interfering with phagosome acidification (6), and scavenging reactive oxygen and/or nitrogen species (7, 8).

Another strategy employed by phagocytic cells to clear infectious agents is through the production of extracellular traps (ETs), consisting of chromatin containing several proteins, commonly derived from intracellular compartments (9). Cells that release ETs following infection include neutrophils, macrophages, eosinophils, basophils, and mast cells (10). These structures have wide antimicrobial activities against many different pathogens including bacteria, protozoa, and fungi (11). Mycobacteria induce ETs formation by neutrophils and macrophages, but curiously, the ETs do not affect bacilli viability (12–14).

Mast cells are particularly abundant in human lungs and are able to detect and respond rapidly to different pathogens (15, 16). In this regard, several studies have shown the importance of mast cells during viral (17), bacterial (18, 19), fungal (20), and protozoan (21) infections. Recognition of bacteria by mast cells leads to release and *de novo* production of inflammatory mediators that recruit effector cells to control the infectious agent (22). However, mast cells also employ diverse mechanism to regulate bacterial growth, including phagocytosis (23), production of antimicrobial peptides (24), and by the production of ETs (MCETs) (25). In this regard, Mtb is able to activate mast cells *in vitro* activating degranulation, inducing the production of inflammatory cytokines, and internalizing bacteria through lipid rafts (26, 27). Moreover, mice treated with a potent inducer of mast cell degranulation C48/80 1 day before Mtb infection showed altered cytokine production and increased lung bacterial loads, suggesting the important protective role of mast cells early during Mtb infection (28).

Considering that mast cells are able to exert antimicrobial activity against both extracellular and intracellular bacteria *via* the release of MCETs (25, 29), here we evaluated whether Mtb induced such structures.

## Materials and Methods

### Bacteria

The bacteria employed in this work were *Staphylococcus aureus* (ATCC 6538), *Mycobacterium tuberculosis* H37Rv (Mtb), and the *katG*-deficient *Mycobacterium tuberculosis* Lehman and Neuman (Mtb KatG−) (ATCC 35822) (30). *S. aureus* was cultured in tryptic soy broth (Dibico, Mexico), while mycobacteria was growth in Middlebrook 7H9 broth (BD-Difco, USA) supplemented with 10% OADC (BD-Difco, USA) and incubated at 37°C in constant shaking at 150 rpm until exponential phase was reached. *S. aureus* inocula were prepared in tryptic soy broth and 10% glycerol, while mycobacteria inocula were done in RPMI-1640 Glutamax (Gibco, USA) supplemented with 10% fetal bovine serum (FBS, Gibco, USA). Bacterial inoculums were adjusted to the McFarland nephelometer No.1 standard tube, corresponding to 3 × 10^8^ bacteria/ml. Bacterial viability was determined after serial dilution and plated in tryptic soy agar (*S. aureus*) or Middlebrook agar (Mtb) and CFU was calculated. Heat-killed Mtb H37Rv (HK-Mtb) was done by incubating Mtb inoculum during 60 min in a water bath at 68°C and bacterial viability was confirmed by seeding in Middlebrook agar.

### Mast Cells

The mast cell line HMC-1 was kindly donated by Dr. J. H. Butterfield, Mayo Clinic, Rochester, MN, USA (31). Cells were growth in RPMI-1640 Glutamax enriched with 10% FBS at 37°C and 5% CO_2_. The cell line was validated by STR DNA fingerprinting by the MD Anderson Cancer Center Characterized Cell Line Core using the AmpFLSTR identifier kit according to the manufacturer’s instructions (Applied Biosystems, Thermo Scientific, Rockford, IL, USA). The STR profiles were compared with known ATCC fingerprints,^1^ to the Cell Line Integrated Molecular Authentication database (CLIMA) version 0.1.200808,^2^ and to the MD Anderson fingerprint database. The STR profiles matched known DNA fingerprints or were unique.

Bone marrow-derived mast cells (BMMC) were obtained as previously described (32, 33). Briefly, bone marrow from the femurs and tibias of 6- to 10-week-old C57BL/6 mice were disaggregated and cultured at a concentration of 1 × 10^6^ cells/ml of RPMI 1640 supplemented with 10% FBS and 10 ng/ml of murine recombinant IL-3 and SCF (Bio-Legend, USA). Non-adherent cells were transferred to fresh culture medium twice a week for 4–8 weeks. Mast cells purity was >90% according to CD117 and FcεRIα measured by flow cytometry (Figure S1 in Supplementary Material). The protocol for BMMC obtainment from mice was reviewed and approved by the Committee for Ethics in Research ENCB, IPN.

### Fluorescent Staining of Extracellular DNA

Extracellular DNA was stained as described (12). In brief, cells were seeded at a density of 5 × 10^5^ cells/ml in RPMI-1640 Glutamax + 2% FBS and seeded on glass coverslips pretreated with 0.001% poly-l-lysine (Sigma Aldrich, USA). Cells were stimulated with 10 MOI of Mtb, KatG−, HK-Mtb, or 25 nM of Phorbol 12-Myristate 13-Acetate or PMA (Sigma Aldrich, USA). Afterward, cells were incubated for different times at 37°C and 5% CO_2_ and fixed with 4% paraformaldehyde (Sigma Aldrich, USA) for 20 min and DNA was stained with 5 µM SYTOX-Green (Molecular Probes, USA). Samples were mounted on a glass slide with Vectashield (Vector Laboratories, USA) and analyzed in a fluorescence microscope Nikon Eclipse E800.

### Extracellular DNA Quantification

Extracellular DNA was quantified as previously described (29). Briefly, MCETs were induced with the different stimuli in RPMI without phenol red + 2% FBS and incubated with 1 U DNase I (Invitrogen, USA) during 30 min. Cell supernatants were collected and 0.5 µM SYTOX-Green (Molecular Probes, USA) was added. Fluorescence was evaluated immediately in a Fluorometer (Fluoroskan Ascent FL, Thermo Scientific, USA) with a filter setting of 485 nm (excitation)/538 nm (emission). Data are presented as increase in the relative fluorescence units (RFU).

### Histone and Tryptase Detection by Immunofluorescence

Cells were seeded on glass coverslips treated with 0.001% poly-l-lysine (Sigma Aldrich, USA) and left unstimulated or stimulated with HK-Mtb (MOI 10) or 25 nM PMA for different times. After incubation at 37°C and 5% CO_2_, cells were fixed with 4% paraformaldehyde and permeabilized with 0.2% Triton X-100 (Sigma Aldrich, USA) during 10 min and blocked with universal blocking reagent (BioGenex, USA). Cells were incubated with either anti-histone H3 (Lifespan Biosciences, USA) or anti-tryptase (Abcam, USA) and revealed with secondary antibody labeled with R-phycoerythrin (histone) (Invitrogen, USA) or Alexa Fluor 488 (tryptase) (Invitrogen USA). DNA was stained with 10 µg/ml 4′,6-Diamidino-2-Phenylindole or DAPI (Invitrogen, USA). Samples were mounted with Vectashield and images were obtained in an Axiovert 200 M confocal microscope (Carl Zeiss, Germany) with 63× oil immersion oil objective. Images were collected and processed with Zeiss LSM Image Pascal version 4.0.

### Evaluation of Antibacterial Activity of MCETs

To induce MCETs, mast cells were stimulated with 25 nM PMA or with HK-Mtb (MOI 10) for 4 h. They then were carefully washed with HBSS (Life Technologies, USA) and replaced with fresh media containing viable *S. aureus* or Mtb (MOI 1) and centrifuged at 400 × *g* for 10 min. The pellet was then incubated at 37°C for 30, 60, 90, 180, and 360 min. After incubation, the bacteria were suspended, the supernatant collected, and CFU was determined. Bacterial survival was determined as percentage in relation to bacteria incubated only in culture media, as previously described (12).

### Evaluation of Hydrogen Peroxide Production

Intracellular hydrogen peroxide was evaluated using a commercial kit (Sigma, USA) following the manufacturer’s instructions. Briefly, 2.5 × 10^5^/100 μl mast cells were plated in a 96-well plate. In some experiments, the cells were pretreated with 0.01 µM diphenyliodonium (DPI) (Sigma, USA) for 30 min to inhibit the NADPH oxidase activity. Cells were left unstimulated, stimulated with 10 MOI of Mtb, KatG−, HK-Mtb, or 25 nM PMA for different times. Data are presented as increase in the RFU.

### Ethical Statement

This study was approved by the Bioethics Committee of Escuela Nacional de Ciencias Biológicas from the Instituto Politécnico Nacional (CEI-ENCB 006/2013).

### Data Analysis and Statics

Data represent the mean ± SD of three independent experiments. Statistical differences between controls and experimental groups were determined using one-way ANOVA followed by the Newman–Keuls method (GraphPad Prism Software V6, USA). A *p*-value <0.05 was considered statically significant.

## Results

### HK-Mtb Induce DNA Release by Mast Cells

**Figure 1:**
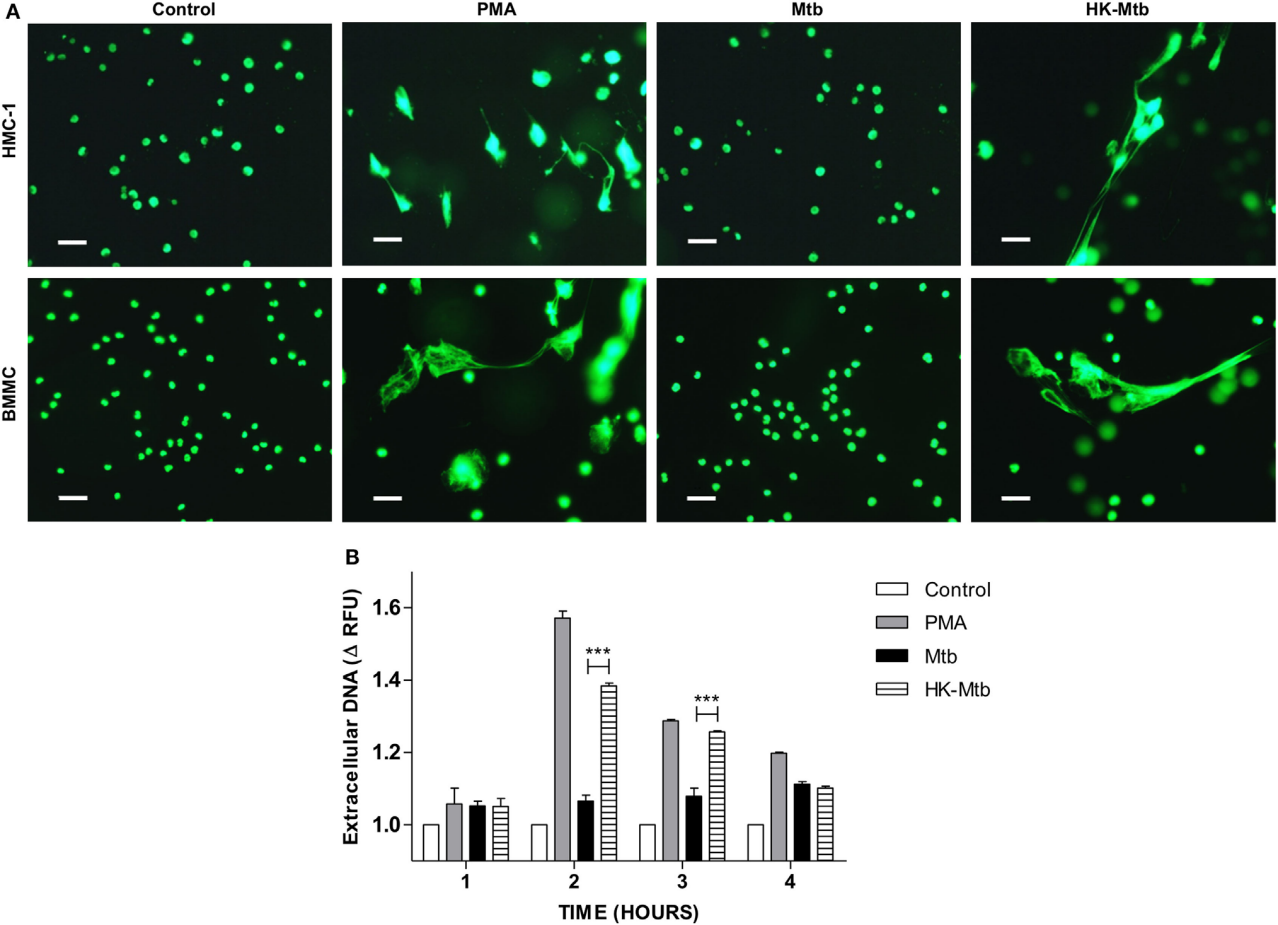
Mast cells release DNA in response to heat-killed but not live *Mycobacterium tuberculosis* (Mtb). **(A)** Representative micrographs of HMC-1 cells or bone marrow-derived mast cells (BMMC) unstimulated (control) or stimulated during 2 h with PMA, live Mtb, or heat-killed Mtb (HK-Mtb) at a MOI of 10. DNA was visualized after staining with SYTOX-Green. Scale bar 20 µm (magnification 400×). **(B)** Released DNA was quantified in unstimulated HMC-1 cells (control) or stimulated at indicated times with PMA, live Mtb, or HK-Mtb at a MOI of 10. Released DNA was partially digested with DNase I and quantified in supernatants with SYTOX Green I in a fluorometer. The graph represents the change in fluorescence ± SD of stimulated cells compared to control. ***p < 0.001 as indicated.

Mast cells are activated by Mtb as evinced by their degranulation, the release of pro-inflammatory cytokines and the ability to internalize bacteria (26, 27). However, whether mast cells are able to release MCETs when activated with Mtb is unknown. To this end, we first evaluated whether Mtb was able to induce DNA release from mast cells at different times. We observed that mast cells, either HMC-1 cells or BMMC, stimulated with PMA released DNA 2-h post-activation (Figure 1A). However, when the mast cells were stimulated with viable Mtb, DNA release was not observed until 4 h of stimulation (Figure 1B). Because viable Mtb employ different strategies to avoid the host immune response, we stimulated mast cells with HK-Mtb to determine if this had any effect. Interestingly, mast cells were able to release DNA after 2 h of stimulation with HK-Mtb (Figures 1A,B), suggesting an active mechanism was employed by Mtb to inhibit the release of DNA. These results show that HK-Mtb, but not live Mtb, is able to induce DNA release by mast cells.

### HK-Mtb Induce MCETs

**Figure 2:**
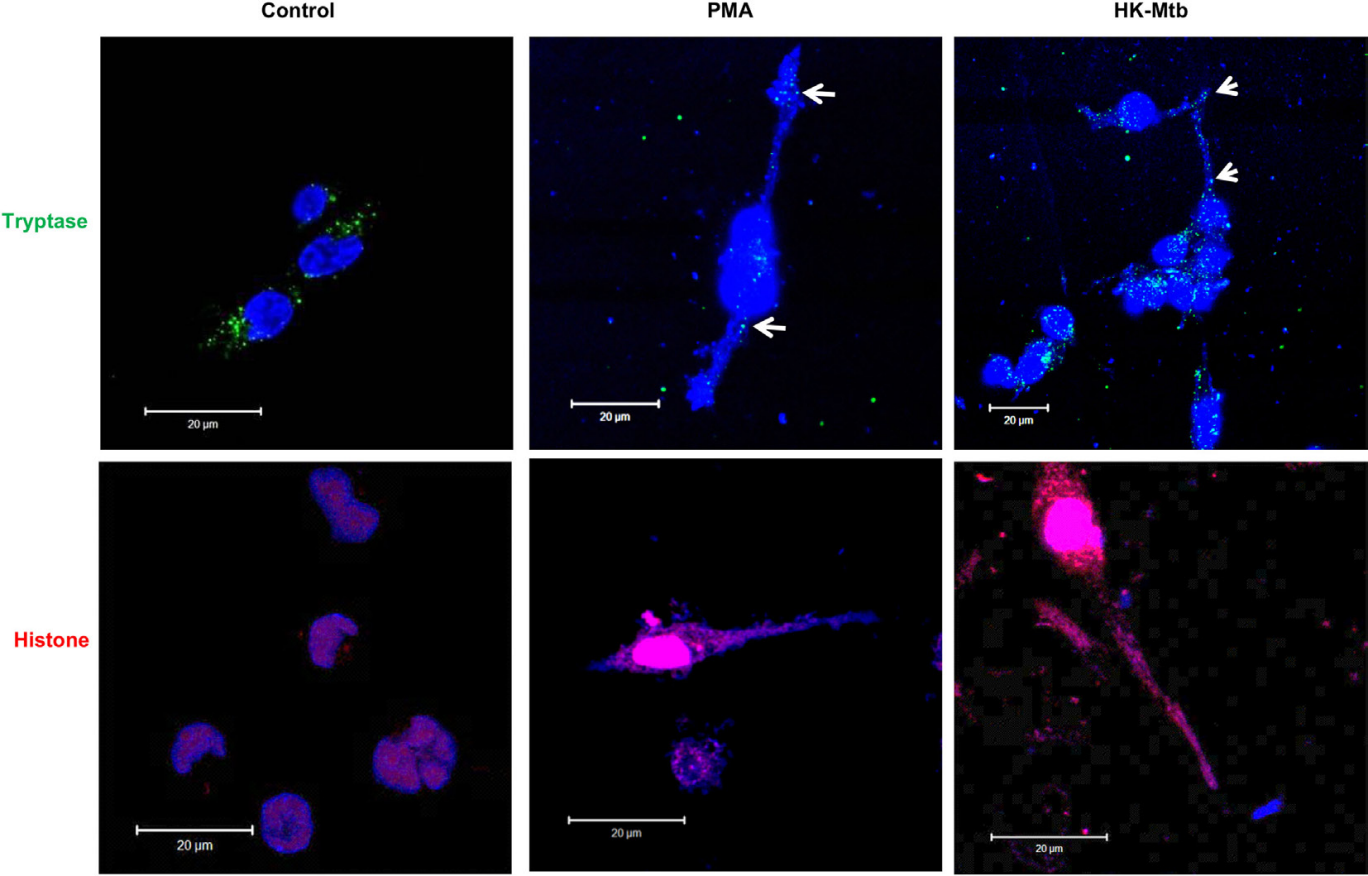
MCETs induced by heat-killed Mtb (HK-Mtb) contain both tryptase and histone. Representative micrographs of unstimulated HMC-1 mast cells (control) or stimulated for 2 h with PMA or HK-Mtb at a MOI of 10. Mast cell tryptase is shown in green, while histone is shown in red and DNA in blue. White arrows indicate DNA zones that showed co-localization with mast cell tryptase. Scale bar 20 µm.

MCETs formation imply the combination of granular proteins of mast cells with nuclear DNA and then its release to the extracellular environment (25). When we analyzed by confocal microscopy the structures released by HK-Mtb-treated mast cells, we observed that extracellular DNA contained both tryptase and histone (Figure 2), indicating that the DNA released by HK-Mtb have components classically found in MCETs.

### Mtb Is Resistant to the Antimicrobial Activity of MCETs

**Figure 3:**
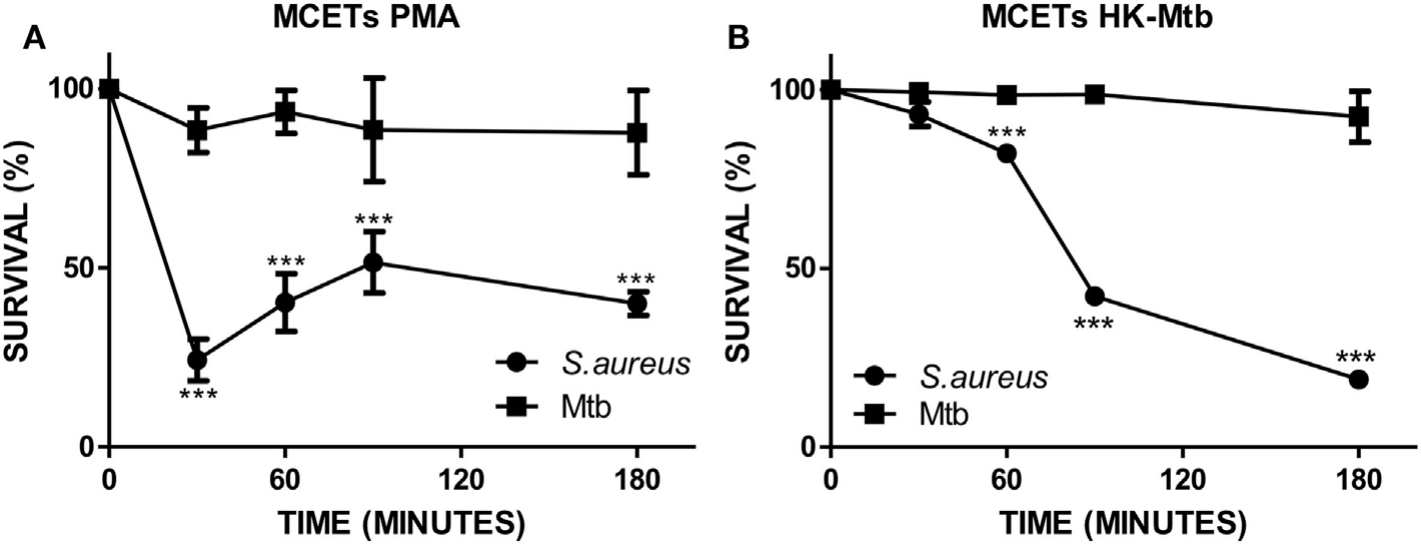
Mast cell extracellular traps do not kill *Mycobacterium tuberculosis* (Mtb). **(A)** HMC-1 cells were activated with PMA or **(B)** heat-killed Mtb (HK-Mtb) to induce MCETs, then Mtb or *Staphylococcus aureus* were added at a MOI of 1 for 3 h Bacteria survival was evaluated by CFU. ***p < 0.001 compared with the initial time.

The main function of MCETs is to ensnare and induce the killing of the entrapped pathogen. Because different signals on mast cells have an impact on the presence of different microbicidal molecules in MCETs (34), we evaluated the ability of induced MCETs with PMA and HK-Mtb to exert antimicrobial activity against viable Mtb. As a control we employed *S. aureus*, because it was previously observed that MCETs showed antimicrobial activity against this bacterium (35). We found that MCETs were able to kill *S. aureus*, but not Mtb, independently if MCETs were induced with PMA or HK-Mtb (Figures 3A,B). These findings indicate that MCETs induced with different stimuli are unable to exert antimicrobial activity against this bacterium.

### Live Mtb Inhibits Hydrogen Peroxide Production by Mast Cells

**Figure 4:**
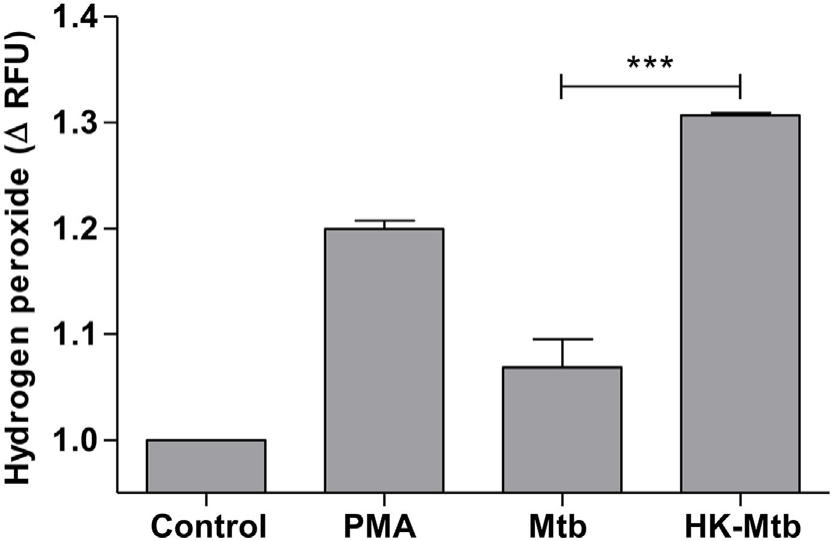
Mast cells stimulated with *Mycobacterium tuberculosis* (Mtb) shows low levels of hydrogen peroxide. HMC-1 cells were left unstimulated (control) or stimulated with PMA, live Mtb, or heat-killed Mtb (HK-Mtb) for 90 min and hydrogen peroxide was evaluated. The graph represents the change in fluorescence ± SD of stimulated cells compared to unstimulated cells. ***p < 0.001 as indicated.

**Figure 5:**
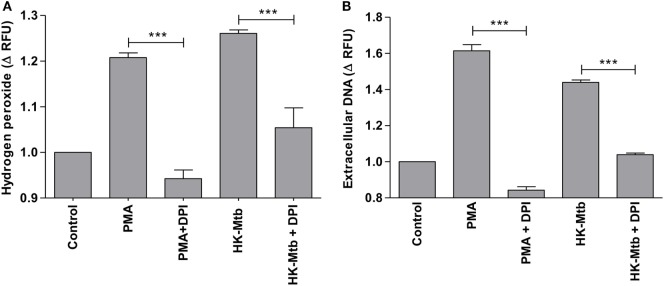
Induction of MCETs by heat-killed Mtb (HK-Mtb) depends on NADPH oxidase. **(A)** Hydrogen peroxide was evaluated in HMC-1 mast cells that were left unstimulated (control) or stimulated for 90 min with PMA or HK-Mtb. A group of cells was incubated with the NADPH oxidase inhibitor, diphenyliodonium (DPI), previous to activation. The graph represents the change in fluorescence ± SD of stimulated cells compared to unstimulated cells. ***p < 0.001 as indicated. **(B)** Extracellular DNA was evaluated in HMC-1 mast cells after 2 h of stimulation with PMA or HK-Mtb in the presence or absence of DPI. The graph represents the change in fluorescence ± SD of stimulated cells compared to the control. ***p < 0.001 as indicated.

Reactive oxygen species (ROS) generated by NADPH oxidase is a crucial step in the induction of MCETs. In particular, the generation of hydrogen peroxide (H_2_O_2_) is considered as a trigger for the release of MCETs (25). Because we previously observed that only HK-Mtb was able to induce MCETs, we evaluated the intracellular production of H_2_O_2_ by live Mtb and HK-Mtb. We noticed that mast cells stimulated with HK-Mtb induced a significant production of intracellular H_2_O_2_ 90 min after stimulation, comparable to that induced by PMA (Figure 4). However, live Mtb was unable to induce a significant amount of H_2_O_2_ at this time (Figure 4). To evaluate whether H_2_O_2_ produced by NADPH oxidase was necessary for the production of MCETs by HK-Mtb we blocked the enzyme activity with DPI. Inhibition of NADPH oxidase activity with DPI significantly diminished H_2_O_2_ production by HK-Mtb and PMA (Figure 5A), and also diminished DNA release by mast cells when compared to control cells stimulated in the absence of DPI (Figure 5B). These results show that H_2_O_2_ produced during the respiratory burst, initiated by NADPH oxidase, is critical for the production of MCETs by HK-Mtb. Moreover, our data suggest that the reduced production of H_2_O_2_ is associated with a poor induction of MCETs.

### Catalase-Deficient Mtb Induce Release of Mast Cell ETs

**Figure 6:**
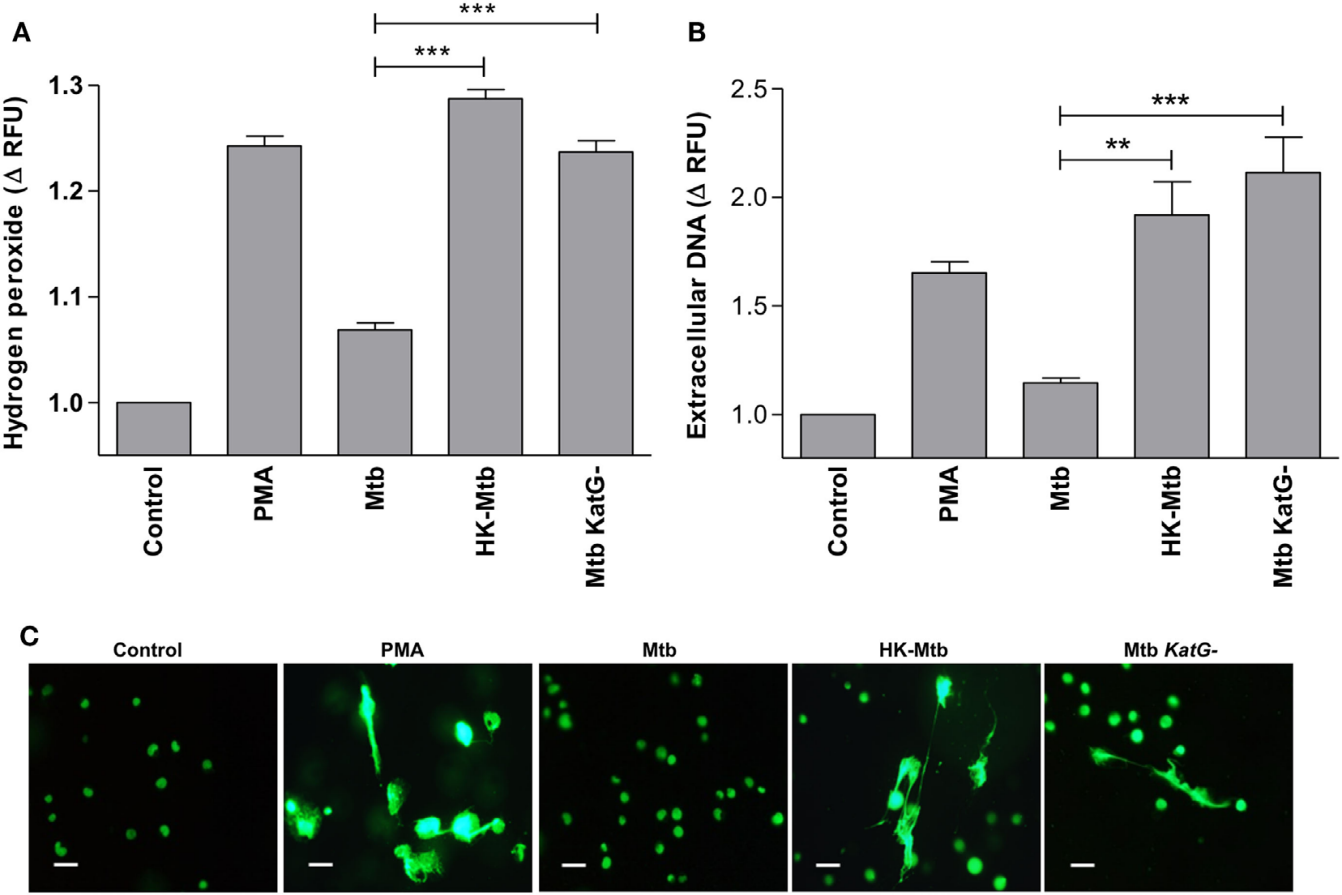
Catalase deficient *Mycobacterium tuberculosis* (Mtb KatG-) induce release of MCETs. **(A)** HMC-1 cells were left unstimulated (Control) or stimulated with PMA, live Mtb, heat-killed Mtb (HK-Mtb), or with a Mtb *katG*-deleted strain (Mtb *KatG−*) for 90 min and H_2_O_2_ was evaluated. The graph represents the change in fluorescence ± SD of stimulated cells compared to unstimulated cells ***p < 0.001 as indicated. **(B)** Extracellular DNA was evaluated in HMC-1 mast cells after 2 h of stimulation with PMA, live Mtb, HK-Mtb, or Mtb *katG−*. The graph represents the change in fluorescence ± SD of stimulated cells compared to the control. ***p < 0.001, **p < 0.01 as indicated. **(C)** Representative micrographs of HMC-1 cells unstimulated (Control) or stimulated during 2 h with PMA, Mtb, HK-Mtb or Mtb *KatG−* at a MOI of 10. DNA was visualized after staining with SYTOX-Green. Scale bar 20 µm (magnification 400×).

*Mycobacterium tuberculosis* overrides ROS produced by cells of the immune system through several ways, one of which implies the presence of genes that code for proteins with catalase activity. It is well known that Mtb strains that develop resistance to isoniazid have deletions in *katG* gene that codes for an enzyme with catalase activity (36). Therefore, we evaluated whether a *katG*-deficient Mtb strain (Mtb *katG−*) was able to induce a significant generation of H_2_O_2_ in mast cells. We observed that mast cells stimulated with Mtb *katG−* had increased levels of H_2_O_2_, comparable to that induced by HK-Mtb and PMA (Figure 6A), and significantly higher than that produced by *katG*+ live Mtb strain (*p* < 0.001). Next, we evaluated the ability of Mtb *katG−* to induce MCETs production. We observed that mast cells stimulated with Mtb *katG−* induced a significant release of DNA, similar to that induced by HK-Mtb or PMA, but significantly higher than that promoted by *katG*+ live Mtb (Figures 6B,C). Taken as a whole, our results indicate that Mtb inhibits the release of MCETs through the production of mycobacterial catalase that activates the decomposition of hydrogen peroxide.

## Discussion

Mast cells develop different functions during an immune response. They play a role in induction of immune regulation, allergy, and can both positively and negatively affect cancer survival, depending upon the type of cancer (16, 32, 33, 37). Of course, one of the main functions of mast cells is to initiate an immune response to different pathogens that surpass the epithelial barrier (22). In this regard, several mechanisms are employed by mast cells to control pathogens, including the induction of a rapid inflammatory response and the development of different antimicrobial activities, such as the production of MCETs. Several pathogens have been identified to induce these structures, including extracellular and intracellular bacteria, fungi, and protozoa (25, 29, 38–40). In this work, we show that Mtb inhibits the release of MCETs in relation to that induced by PMA and other intracellular bacteria, such as *Listeria monocytogenes* (29). Our findings indicate that mycobacterial catalase encoded by *katG* plays an essential role in the ability of this organism to evade the immune response through the decomposition of hydrogen peroxide that is an essential trigger for MCETs induction (25). To the best of our knowledge, this represents a novel, so far unrecognized strategy employed by pathogens to override ETs.

One of the classical mechanisms identified to evade ETs is by the presence of pathogen-derived nucleases, which dismantle the DNA backbone of ETs. This phenomenon was observed during the infection with *S. aureus*, whose nuclease is able to degrade neutrophil extracellular traps (NETs), generating deoxyadenosine that exert toxic effects in infiltrating macrophages (41). A different identified mechanism is by interfering with the activation of the immune cells that produce ETs. For instance, Group A *Streptococcus* expresses a high-molecular weight hyaluronan that engages Siglec-9 in human neutrophils that blocks the cell signaling that leads to ROS generation and NETs formation (42). On the other hand, *Acinetobacter baumannii* inhibits NET formation by altering neutrophil adhesion by reducing CD11a expression on neutrophils (43), while the capsular polysaccharides glucoronoxylomanan from *Cryptococcus neoformans* inhibit NET production by blocking ROS production (44).

Hydrogen peroxide is an essential molecule in the formation of MCETs (25), and several pathogens promote the degradation of this molecule through the presence of genes that code for enzymes with catalase activity (45). In this work, we observed that HK-Mtb, but not viable Mtb, was able to induce the release of MCETs. Moreover, we noticed that HK-Mtb induced significant levels of hydrogen peroxide, that were diminished in mast cells stimulated with live Mtb, indicating that live Mtb targeted production of this molecule. We decided to evaluate if mycobacterial *katG* was involved in this blockade because of two reasons: (1) it codes for an enzyme with the ability to decompose hydrogen peroxide into water and molecular oxygen and (2) the deletion of this gene usually occur in Mtb strains that develop resistance to isoniazid, but are less virulent in guinea pig model of infection (36). Our findings indicate that mast cells stimulated with live Mtb, in which *katG* was deleted, showed an increased accumulation of hydrogen peroxide and were able to induce the release of MCETs. These results show that *katG* helps mycobacteria to override MCETs production by depressing mast cell production of hydrogen peroxide, thus suppressing a mechanism that triggers MCETs formation.

Because Mtb inhibited MCETs formation it seems logical that this is one mechanism by which Mtb avoids the antibacterial activity of such structures. To test this hypothesis, we induced MCETs with PMA or HK-Mtb and incubated them with viable Mtb to evaluate their antibacterial activity. We noticed that although such MCETs were able to exert antimicrobial activity against *S. aureus*, they did not affect mycobacterial viability. The resistance of Mtb to ETs is also observed with those released by neutrophils and macrophages (12, 13). This is an interesting observation because although the main effect associated with antimicrobial activity in ETs is related to the presence of antimicrobial peptides, the presence of specific granule enzymes also contributes to antimicrobial activity (29), and Mtb is resistant to the antimicrobial activity of the different components present in the different ETs of macrophages, neutrophils, and mast cells. This is probably due to the particular characteristics of the cell wall of Mtb, although the components that are implied in this process need to be clarified. In this regard, resistance to the antimicrobial activity is described in other pathogens such as *Streptococcus pneumoniae* that alters lipoteichoic acid by the incorporation of d-alanine rendering resistance to the bactericidal activity of NETs, while *Cryptococcus gattii* produce extracellular fibrils that render resistance to NETs (46, 47).

A second possibility is that Mtb blocks MCETs formation to avoid the death of mast cells and that these cells exert an inflammatory process that favors infection. It is well known that the inflammatory process during the early phase of mycobacterial infection is crucial for the recruitment of macrophages that are susceptible to the infection and promote the spread to other tissues (48). On the other hand, mast cells release preformed inflammatory mediators and induce the production of pro-inflammatory cytokines when stimulated with Mtb (26). However, further work is needed to clarify the role of the blockade of MCETs production during Mtb infection.

Finally, one limitation of our work is that although we used two different sources of mast cells with different levels of maturity, we did not employ a completely mature mast cell that represent the phenotype of lung mast cells. It is well known that mast cells have a great heterogeneity in their phenotype and function depending on the tissue they reside (49), so further work is needed to clarify this issue.

In conclusion, our work unravels a previously unrecognized mechanism employed by Mtb to override the immune response through the inhibition of MCETs production, employing *katG* to decompose hydrogen peroxide. We hypothesize that MCETs blockade by this bacterium could be relevant during the early phase of the infection.

## Ethics Statement

This study was approved by the Bioethics Committee of Escuela Nacional de Ciencias Biológicas from the Instituto Politécnico Nacional (CEI-ENCB 006/2013).

## Author Contributions

MC-N, KL-P, LD-M, GR-L, and RS-C performed experiments and analyzed data. MC-N, KL-P, LD-M, BG-P, LF-R, SE-P, IE-G, and RC-S analyzed and interpreted data. MC-N, NP-O, SU, JL-H, LF-R, HS-L, SP-T, and RC-S interpreted data, drafted the manuscript, and contributed with intellectual content. RC-S designed, supervised the study, and obtained funding. All the authors critically revised and approved the final version of this manuscript.

## Conflict of Interest Statement

The authors declare that the research was conducted in the absence of any commercial or financial relationships that could be construed as a potential conflict of interest.

## Footnotes

^1^https://ATCC.org (Accessed: December, 2017).

^2^http://bioinformatics.hsanmartino.it/clima/ (Accessed: December, 2017).

## Supplementary Material

Figure S1 Bone marrow-derived mast cell purity. **(A)** Representative flow cytometry density plot of bone marrow cells after 6 weeks of culture with IL-3 and SCF. The percentage of FcεRI+CD117+ cells is shown. **(B)** BMMC were stained with toluidine blue and analyzed by light microscopy (magnification 1,000×).
