# Supplementary figures and images for: Mycobacterium tuberculosis Catalase Inhibits the Formation of Mast Cell Extracellular Traps
Source: Front Immunol. 2018 May 28;9:1161. doi: 10.3389/fimmu.2018.01161 (PMC5985745; doi:10.3389/fimmu.2018.01161)

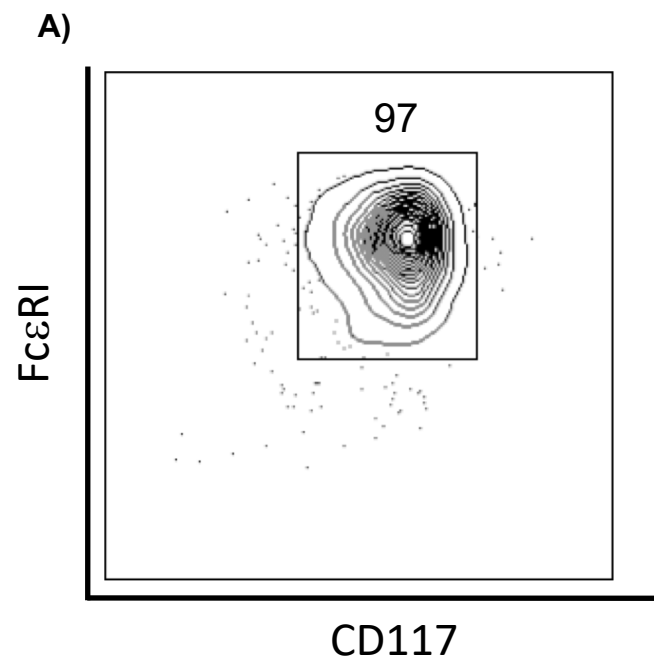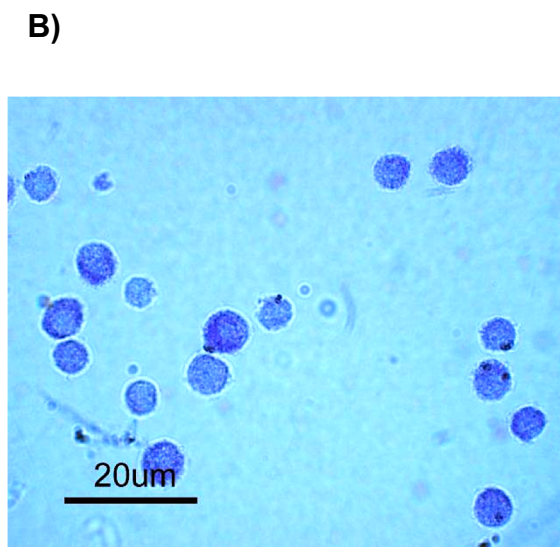

Supplement: Figure S1 — Bone marrow-derived mast cell purity. (A) Representative flow cytometry density plot of bone marrow cells after 6 weeks of culture with IL-3 and SCF. The percentage of FcεRI+CD117+ cells is shown. (B) BMMC were stained with toluidine blue and analyzed by light microscopy (magnification 1,000×). [file Image_1.PDF]
